# Research skills: the neglected competency in tomorrow’s 21st-century doctors

**DOI:** 10.1007/s40037-013-0087-7

**Published:** 2013-10-04

**Authors:** Ahmed Abu-Zaid

**Affiliations:** College of Medicine, Alfaisal University, P.O. Box 50927, Riyadh, 11533 Saudi Arabia

Lemon and colleagues [1] published an interesting article shedding light on an issue which I call: ‘research skills: the neglected competency in tomorrow’s 21st century doctors’. The authors asserted the unrecognized compulsoriness of research skills for medical undergraduates [1]. This perspective has been also emphasized by the Boyer Commission on Educating Undergraduates in the Research University [2].

Few medical students express interest in undergraduate research engagement and research-focused careers. The reasons for this include: insufficient exposure to scientific research early in education, no interest in incorporating a research component into education, unwillingness to prolong medical training, personal preference for direct clinical practice-based careers, and failure to realize what it really means to pursue research-focused physician-scientist or academic/clinical medicine careers before a career decision is determined.

Generating research-oriented medical workforces is necessary. Intentions of medical students in undertaking research activities may be wheeled by aspirations to develop research-based competencies, curiosities to explore particular scientific disciplines, or pure strategic tactics to smooth acceptance to residency, fellowship and other higher degree medical programmes of choice. However, regardless of the intentions, it is crucial to guide students to develop positive attitudes towards scientific research as the substratum for modern medicine. Also, it must be conveyed that possessing a solid knowledge base and skills in scientific research is becoming an essential competency for tomorrow’s 21st century doctors. Moreover, it must be pointed out that practising biomedical/clinical scientific research is indispensible to up-to-date evidence-based medicine, incompletely distanced from clinical practice and directly contributes to patient care. Furthermore, it is essential to illuminate the significance of physician-scientists and their important contributions to translational research—the primary driving force towards improving patient well-being by promoting the ‘bench-to-bedside’ transition [3].

Teaching scientific research (theoretically and practically), and engagement in research endeavours—early in medical education—enhances research intellectual and practical skills, nurtures high-order cognitive skills (e.g., critical appraising, problem troubleshooting, idea processing and wise judging), augments interest in inquiry-based learning, generates scientific publications, encourages involvement in future research activities and ultimately supports entry to varying research-focused careers.

Moreover, students’ development of positive attitudes towards research and future research professions are considerably influenced by the presence of enthusiastic research-oriented teachers who possess a vast research expertise and skillful teaching capabilities. Those teachers—as passionate instructors, ideal role models, valuable mentors and ambassadors of scientific research—are anticipated to spike intensified interest of students in research, illuminate its indispensable relevance in contemporary medicine, encourage research-focused careers and resolve all research-related obstacles standing in the face of students. Unfortunately, such researchers/teachers are not present in many medical schools and universities—an issue to be highly reconsidered by medical education councils.

It is possibly the right time to explore undergraduates’ perceptions of the so-called: ‘research skills for undergraduates: a must’ [1] as to acquire a thorough understanding of the philosophical viewpoint: ‘teaching scientific research is indispensable in the 21st century undergraduate medical curricula’. The evolving trend towards utilizing students’ perceptions is largely driven by the universal move towards student-centred education. Students’ perceptions represent valuable inputs to effectively address curricular concerns and accordingly optimize medical education. Lastly, I would like to praise the authors and congratulate the editors for bringing into our attention this endlessly important topic for further continued discussion.
